# T‐cell exhaustion in HIV infection

**DOI:** 10.1111/imr.12823

**Published:** 2019-12-27

**Authors:** Craig Fenwick, Victor Joo, Patricia Jacquier, Alessandra Noto, Riddhima Banga, Matthieu Perreau, Giuseppe Pantaleo

**Affiliations:** ^1^ Service of Immunology and Allergy Department of Medicine Lausanne University Hospital University of Lausanne Lausanne Switzerland; ^2^ Swiss Vaccine Research Institute Lausanne University Hospital University of Lausanne Lausanne Switzerland

**Keywords:** chronic viral infection, HIV, immune checkpoint inhibitors, PD‐1, T‐cell exhaustion

## Abstract

The T‐cell response is central in the adaptive immune‐mediated elimination of pathogen‐infected and/or cancer cells. This activated T‐cell response can inflict an overwhelming degree of damage to the targeted cells, which in most instances leads to the control and elimination of foreign invaders. However, in conditions of chronic infection, persistent exposure of T cells to high levels of antigen results in a severe T‐cell dysfunctional state called exhaustion. T‐cell exhaustion leads to a suboptimal immune‐mediated control of multiple viral infections including the human immunodeficiency virus (HIV). In this review, we will discuss the role of T‐cell exhaustion in HIV disease progression, the long‐term defect of T‐cell function even in aviremic patients on antiretroviral therapy (ART), the role of exhaustion‐specific markers in maintaining a reservoir of latently infected cells, and exploiting these markers in HIV cure strategies.

## 1 GENERAL INTRODUCTION ON T‐CELL EXHAUSTION

The T‐cell activation paradigm proceeds in a highly organized process involving three signals consisting of antigen recognition, receptor costimulation, and a termination signal that are required for the tight regulation of a strong functional and proliferative response.

Signal one in T‐cell activation represents the specific recognition through the T‐cell receptor (TCR) of their cognate antigen presented by professional antigen‐presenting cells (APCs) including dendritic cells, macrophages, and B cells.

Signal two is the costimulatory signal where receptors on the T cells bind to their counterpart ligands. CD28 is the primary costimulatory receptor for T cells that acts through interaction with its ligands, CD80 and CD86 expressed on APCs. Costimulation is essential for T‐cell activation since signal one TCR/antigen recognition in the absence of signal two drives the cell toward an anergic and/or tolerogenic state.^1^ Additional costimulatory receptors that can enhance T‐cell activation include CD27, OX40 (CD134), ICOS (CD278), CD40L (CD154), CD226 that bind to CD70, OX40L (CD252), B7‐H2 (CD275), CD40, and CD155/CD112, respectively, on APCs.^2^ The concentration of costimulatory and ligand molecules can vary significantly such that either no positive signal is sent or a paired interaction provides a strong supporting signal two to the T cells. Soluble pro‐inflammatory cytokines including IL‐12 and type I interferons contribute to a fully activated T‐cell response.

Signal three arises in the days following T‐cell activation, the effector phase of the immune response, and eventually the elimination of the pathogen. Signal three is responsible for terminating the immune response. Inhibitory receptors, also called immune checkpoint inhibitors (ICIs), exert their influence on T‐cell activation at this stage where under normal conditions, ICIs including programmed cell death receptor 1 (PD‐1), cytotoxic T lymphocyte antigen‐4 (CTLA‐4), LAG3 (lymphocyte activation gene protein), and TIM3 (T‐cell immunoglobulin domain and mucin domain‐containing protein 3) are transiently upregulated on the surface of effector T cells within hours to days following T‐cell activation. Here, their purpose is to attenuate T‐cell activation and limit immune function at the end stages of an acute infection when a pathogen is controlled. Upon clearance of antigen, the expression of these ICIs on memory T cells declines to normal levels over time. Termination of the T‐cell response is an essential self‐limiting mechanisms to help preserve self‐tolerance. However, in the case of chronic infection or cancer when the immune response is incapable of clearing the foreign antigen and there persistent chronic stimulation, T cells can enter a dysfunctional state called exhaustion.

**Figure 1:**
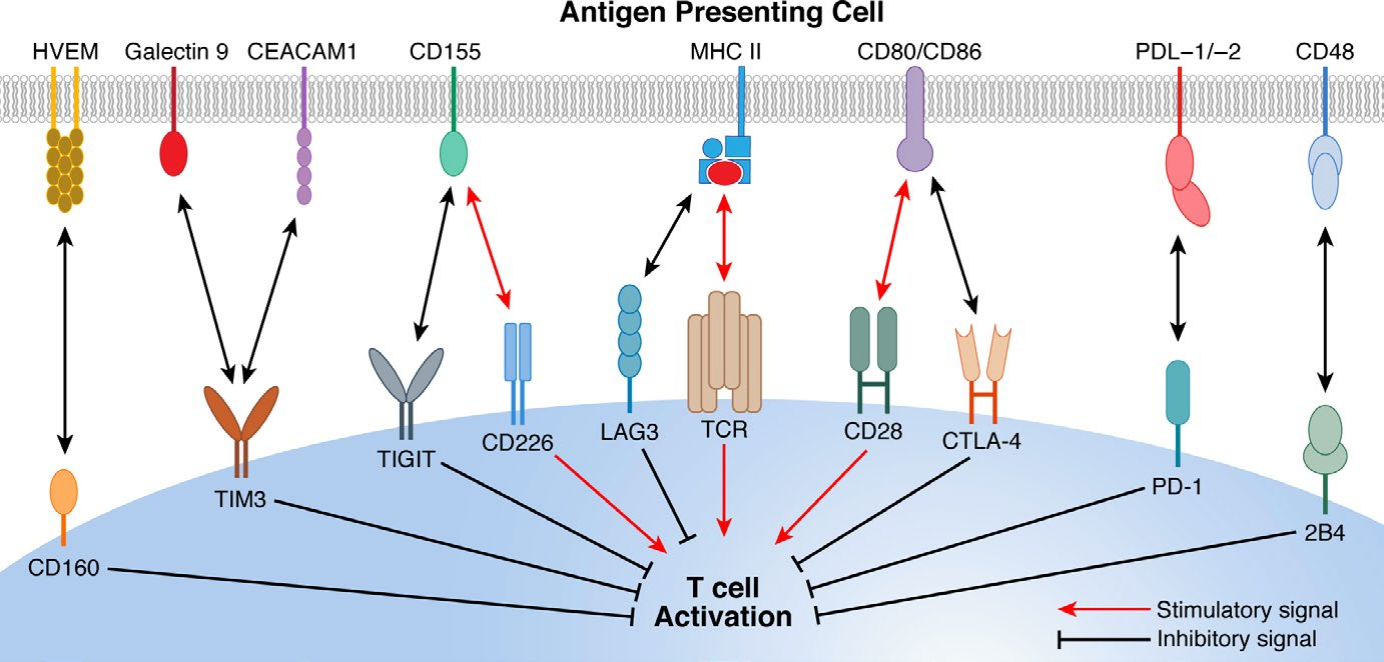
T‐cell immune checkpoint inhibitors and related ligands. TCR interaction with the antigenic peptide‐MHC complex displayed by professional APCs delivers the primary signal for T‐cell activation. The CD28 co‐receptor and other costimulatory receptors enhance the T‐cell stimulatory signal following interaction with their corresponding ligands. Immune checkpoint inhibitors including PD‐1, CTLA‐4, LAG3, TIGIT, TIM3, CD160, and 2B4 act to suppress T‐cell signal following interaction with their related ligands expressed on APCs

T‐cell exhaustion is a progressive condition with increasing loss in effector function coinciding with increased expression levels and assortment of ICIs. PD‐1 is recognized as the master inhibitory regulator of T‐cell function but with increased degrees of exhaustion comes elevated levels of additional ICIs including CTLA‐4, TIM3, LAG3, T‐cell immunoreceptor with Ig and ITIM domains (TIGIT), 2B4 (CD244), and CD160^3^, ^4^ (Figure 1). T‐cell exhaustion mediated through PD‐1 relies on interaction with its cognate ligands, PDL‐1 and PD‐L2, which regulate the delicate balance between immune defense and the protection of healthy tissue. Immune cells, non‐immune endothelial, and epithelial cells constitutively express PDL‐1, while PDL‐2 expression is limited to APCs. The expression of PDL‐1 is further upregulated after activation, which modulates the immune responses against self and foreign antigens.^5^ In a likewise fashion, the interaction of additional ICIs with their respective ligands exert a further level of control and suppression of T‐cell function.

## 2 FUNCTIONAL PROFILE

In exhausted CD8 T cells, the increased expression of ICIs leads to a hierarchical loss of function that begins with reduced IL‐2 secretion, cytokine polyfunctionality, and diminished proliferative potential. Functional defects progress to the loss in cytotoxic capacity and TNF‐α secretion with highly exhausted cells having reduced ability to produce IFN‐γ.^6^, ^7^, ^8^ Although T‐cell exhaustion was first identified in CD8 T cells, it is now accepted that CD4 T cells are also subject to exhaustion leading to reduced production of IL‐2, IFN‐γ, and TNF‐α^9^, ^10^, ^11^ along with reduced CD4 T‐cell help.^12^ In addition to persistent antigen and pathogen burden, the loss or reduction of CD4 T‐cell help is an important factor that contributes to the initial establishment of exhaustion.^13^, ^14^, ^15^ The precise nature of optimal CD4 T‐cell help needed to antagonize exhaustion is unclear, but is likely to involve the production of IL‐2 and IL‐21 cytokines that support CD8 T‐cell response both directly and indirectly through activation of APCs.^16^

Aside from a blunted immune response against infected or cancerous cells, exhausted T cells have a poor or varied response to homeostatic cytokines including IL‐7 and IL‐15 responsible for the maintenance of memory T cells. In the case of complete clearance of a viral pathogen or in adoptive transfer studies to antigen‐free mice, exhausted T cells are poorly maintained through self‐renewal.^17^, ^18^, ^19^ Instead, persistence of exhausted T cells occurs through continual antigen signals that promotes proliferation.^20^ Exhausted T cells can persist in vivo for years but in the final stages of exhaustion with high antigen stimulation, there is a loss of the virus or tumor‐specific cells through apoptosis.^7^, ^21^ Further characteristics of T‐cell exhaustion include altered transcription factor expression, metabolic profile and epigenetic modifications that are distinct from other memory T‐cell subsets.

## 3 TRANSCRIPTIONAL PROFILE

The transcriptional profile of exhausted CD4 and CD8 T cells are significantly different from all memory T‐cell subsets. Although several transcriptional factors are correlated with exhausted T cells including NFAT, Batf, IRF‐4, T‐bet, Eomes, and Blimp‐1, no master regulator of exhaustion has been identified. NFAT activation during chronic infection along with a corresponding low nuclear translocation of AP‐1 induces strong transcriptional activation of ICIs including PD‐1, LAG3, and Tim3.^22^, ^23^, ^24^, ^25^ Signaling through the PD‐1 receptor induces the upregulation of the Batf transcription factor, which in turn inhibits AP‐1 activation and contributes to the sustained high levels of PD‐1. Indeed, exhausted CD8 T cells from chronically infected HIV donors have elevated levels of Batf with higher levels observed in patients that experience disease progression compared to those that spontaneously controlled their HIV viral loads.^24^ In the chronic lymphocytic choriomeningitis virus (LCMV) mouse infection model, IRF‐4 contributes to T‐cell exhaustion in consort with Batf and NFAT and reduction of IRF‐4 expression restores the functional properties of exhausted antigen‐specific T cells.^26^ T‐bet and Eomes transcription factors are individually important for the development of KLRG‐1+ terminal effector CD8 T cells in response to inflammation and the maintenance of memory cells, respectively. In contrast, both are essential for the development of exhaustion as demonstrated in studies involving the genetic deletion of either^20^ and both are upregulated in exhausted T cells with different viral infections.^27^ Although necessary for exhaustion, high levels of the T‐bet directly repress the transcription of the PD‐1 gene. It is therefore interesting that in combined expression with Eomes, the T‐bet^high^ Eomes^dim^ population represent an intermediate exhausted subset with progenitor capacity and a sustained virus‐specific CD8 T‐cell response during chronic infection.^28^, ^29^ In HIV‐infected donors, exhausted T cells with a skewed balance toward T‐bet^dim^ Eomes^high^ expressional profile represent a highly functionally exhausted state with elevated levels of multiple ICIs including PD‐1, CD160, and 2B4 on CD8 T cells.^30^, ^31^, ^32^ Blimp‐1 is a primary transcription factor for the differentiation of germinal center B cells; however, it is also upregulated in exhausted T cells and correlates with the protein expression levels of ICIs. Similar to Batf, Blimp‐1 is upregulated in progressor patients with chronic HIV infection compared to non‐progressors that control HIV viral loads.^33^ Blimp‐1 expression in CD4 T cells mediates the production of IL‐10, which can further contribute to the dysfunctional state of exhausted T cells during chronic viral infection.^34^ Exhausted T cells may coexpression pairs of transcription factors including Blimp‐1 and Eomes, however, expression is not uniform where combinations of different factors may coexist. Therefore, along with the phenotypic and functional profiles, exhausted T cells consist of a heterogeneous population with varied expression of transcription factors.^12^, ^22^, ^35^ This diversity of exhausted T‐cell populations is exemplified in a recent study using high dimensional mass cytometry to analyze cells from chronic HIV‐infected donors and in human tumors. Using phenotypic, functional, transcription factor, and ICI coexpression patterns, nine distinct exhausted CD8 T‐clustered cell populations were identified.^36^

## 4 METABOLIC ABNORMALITIES AND EXHAUSTION

The metabolic profile of T cells shifts from the use of the oxidative phosphorylation pathway in naive cells to glycolysis in the acute phase of an infection when activated effector cells have increased bioenergetics needs. Once the infection is cleared, memory cells revert to a quiescent state that uses oxidative phosphorylation and gains the additional metabolic ability for fatty acid oxidation.^37^ With chronic antigen stimulation and the onset of exhaustion, the suppression of the glycolysis pathway ensues with reduced cellular glucose uptake and signs of a dysregulated mitochondrial function.^38^ The utilization of endogenous fatty acids by exhausted cells may dictate the available energy reserves under conditions of ICIs engagement.^39^ Metabolic pathways implicated in this defective state include transcriptions control through Foxo1^40^ and PGC1α.^41^

## 5 EPIGENETICS AND EXHAUSTION

Epigenetic modifications associated with T‐cell exhaustion begins within the first 2‐3 weeks of chronic LCMV infection. This programming is irreversible, even in adoptively transferred studies with uninfected antigen‐free mice where T cells maintain their exhausted transcriptional and functional profile.^12^, ^27^, ^42^ The epigenetic landscape of exhaustion has been studied by comparing CD8 T cells during acute and chronic phases of LCMV infection. Changes consisted in large reorganizations of chromatin‐accessible regions resulting in altered access to transcriptional start sites. These modifications were associated with the induction of multiple genes including Pdcd1 (PD‐1), Havcr2 (Tim3), and Batf which are known to be upregulated in exhausted CD8 T cells. The epigenetic modifications in Pdcd1 include histone acetylation in the promoter and proximal enhancer region followed by full demethylation in these regions that is maintained in exhausted cells and allows for consistent high PD‐1 expression. These epigenetic changes are also present in several chronic viral infections including HIV, CMV, and EBV.^43^ Indeed, the vast majority of this epigenetic program linked to exhaustion in mice (approximately 80%) was also successfully mapped to tetramer positive CD8 T cells of treatment naive chronically infected HIV donors.^44^, ^45^, ^46^ As such, epigenetic modifications play a key role in maintaining T cells in an exhausted state.

## 6 T‐CELL EXHAUSTION IN HIV INFECTION (CAUSES OF EXHAUSTION, PHENOTYPIC MARKERS OF EXHAUSTION, AND FUNCTIONAL EXHAUSTION)

T‐cell exhaustion was first described in mouse models with chronic LCMV infection where antigen‐specific CD8 T cells progressively lost their effector functions and developed a reduced capacity to kill virally infected cells.^47^, ^48^, ^49^, ^50^ Subsequent to these studies, it became clear that the same principles of T‐cell exhaustion occurred in human chronic viral infections including HIV, HCV, HBV, and HTLV‐1 as well as cancer.

Chronic HIV infection occurs in most patients in the presence of persistent high levels of virus replication and is associated with a loss of immune control of virus replication. Virus‐specific CD8 T cells partially suppress HIV viral replication in the initial stages of infection. In a similar manner, SIV‐infected macaque studies showed that CD8 T‐cell depletion leads to a dramatic increase in viral load.^51^ However, with persistent high levels of viral antigen, HIV‐specific T cells become exhausted and lose their capacity to kill efficiently infected cells. In addition to high levels of viral antigen, the strong pro‐inflammatory immune activation during HIV infection and a compromised T‐cell homeostasis during HIV infection contribute to the development of T‐cell exhaustion.^52^, ^53^

**Figure 2:**
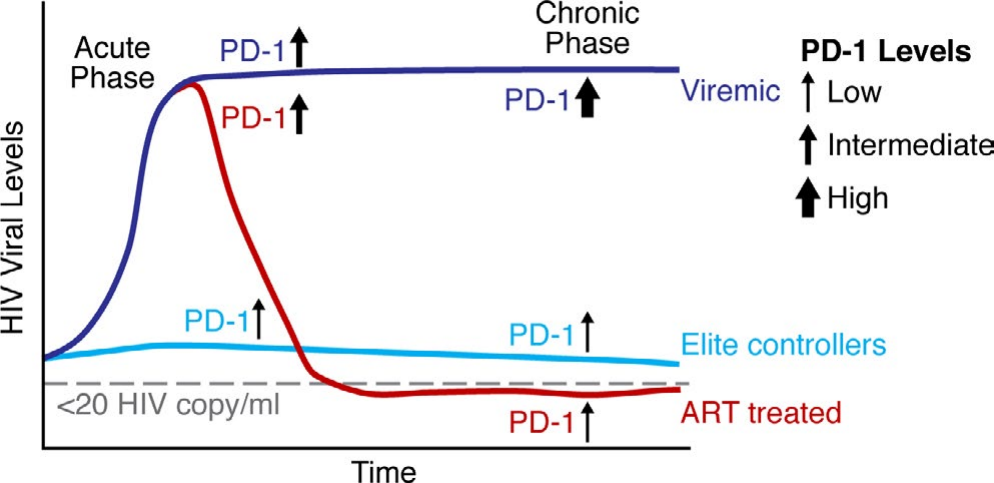
HIV Infection and progression of exhaustion. During the acute phase of HIV infection, the majority of individuals experience a dramatic increase in HIV viral load and increased levels of the PD‐1 receptor on HIV‐specific T cells. In contrast, elite controllers maintain low viral load levels and consequently, T cells express low levels of PD‐1. Untreated patients with high viral load progress to the chronic phase of HIV infection where persistent elevated levels of HIV antigen result in T‐cell exhaustion with high expression levels of PD‐1. ART in most patients significantly inhibits viral replication, resulting in decreased plasma viral load to levels below the limit of detection with standard assays. With low levels of viral antigen present, PD‐1 expression decreases to lower levels on HIV‐specific T cells

**Figure 3:**
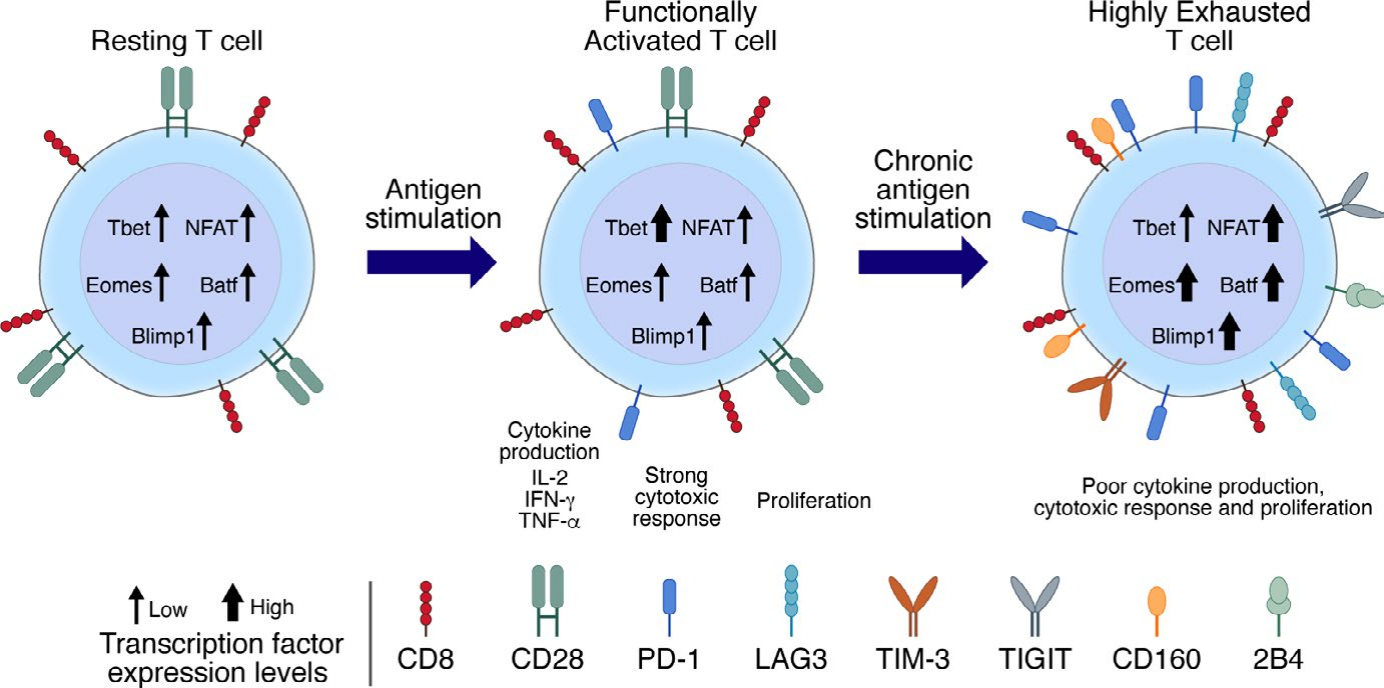
Phenotype, transcriptional, and functional profile of T cells progressing to exhaustion. Antigen‐specific stimulation of resting T cells leads to a functionally active T‐cell response with increased cell surface PD‐1, upregulated expression of the T‐bet transcription factor and a strong functional and proliferative response. Chronic stimulation with high levels of antigen drive T cells into an exhausted state characterized by high levels of PD‐1, an increased expression of additional immune checkpoint inhibitors and a pronounced T‐cell dysfunction. Compared to functionally active T cells, highly exhausted T cells have elevated expression of transcription factors including NFAT, Batf, Eomes, and Blimp‐1 with decreased levels of T‐bet

In HIV‐1 infection, PD‐1 expression on virus‐specific T cells is the primary marker of exhaustion that correlates with disease progression. Pivotal studies showed that PD‐1 expression correlated with impairment of CD8 T cells functionality, viral load, and reduced the CD4 T‐cell counts.^54^, ^55^, ^56^ Importantly, cytomegalovirus‐specific CD8 T cells from the same HIV‐infected donors did not upregulate PD‐1 and preserved the functional capacity to produced high levels of cytokines. This demonstrated that the T‐cell defects were HIV‐specific and driven primarily by the high level of antigen that induced exhaustion. Longitudinal studies show that following ART initiation, PD‐1 expression levels gradually decrease on HIV‐specific CD8 T cells. Long‐term non‐progressors (LTNPs) or viremic controllers were also evaluated and show lower levels of PD‐1 expression on HIV‐specific T cells that exhibited a stronger effector functions as compared to progressors^54^, ^55^ (Figure 2). Aside from PD‐1, other ICIs including LAG3, Tim3, TIGIT, 2B4, and CD160 expressed independently or combined lead to more pronounced stages of CD8 T‐cell exhaustion.^54^, ^57^, ^58^, ^59^, ^60^, ^61^, ^62^ Coexpression of PD‐1 with TIGIT was shown to correlate with disease progression in both HIV‐infected patients and SIV infection model.^58^ PD‐1 and LAG3 expression either alone or in combination with CD38 expression also correlates with the with plasma viral load of patients and was predictive of the time to disease progression.^63^ Similarly, simultaneous expression of PD‐1, CD160, 2B4, and LAG3 on CD8 T‐cell populations correlated directly with HIV load and inversely with the multiplicity of functional outputs exhibited by HIV‐specific CD8 T cells^64^ (Figure 3).

The progressive loss of CD8 T‐cell function starts with an initial loss of proliferative capacity, cytotoxic potential, and a restricted IL‐2 production. In more pronounced stages of exhaustion associated with chronic exposure of T cells to viral antigen, T cells eventually lose the ability to produce IFN‐γ.^55^, ^56^, ^65^, ^66^ HIV‐specific CD8 T cells with elevated levels of PD‐1 also show greater susceptible to apoptosis that was attributed to lower levels of the pro‐survival Bcl‐2 and higher levels of CD95/Fas surface receptor compared to the PD‐1 low T cells.^67^

The focus of T‐cell exhaustion is often on the loss of CD8 T cells function that is primarily responsible for the killing infected cells. However, CD4 helper T cells also exhibit functional defects during HIV infection. Exhausted CD4 T cells exhibit a reduced HIV‐specific proliferative capacity and a loss in polyfunctional cytokine response that centers on reduced IL‐2 production.^68^, ^69^ Exhausted virus‐specific CD4 T cells also express PD‐1 with elevated levels correlating with disease progression, viral loads and reduced CD4 T‐cell count.^70^ As such, PD‐1 is a common regulator of exhaustion on HIV‐specific CD4 and CD8 T cells. In contrast, the CTLA‐4 ICI is more selectively upregulated on exhausted CD4 T cells that correlates with disease progression and T‐cell dysfunction.^71^ Conversely, the ICIs 2B4 and CD160 that are characteristically upregulated on exhausted CD8 T cells are virtually absent from exhausted CD4 T cells.^72^ As with CD8 T cells, expression of multiple ICIs including PD‐1, CTLA‐4, and TIM3 is associated with a more pronounced state of functional CD4 T‐cell exhaustion.^73^ A characteristic feature of CD4 and CD8 exhausted T cells is both experience at least partial restoration of antigen‐specific proliferative and functional activity following antibody‐mediated ICI blockade therapy.

Aside from increased levels of ICIs during HIV infection, an activated T‐cell phenotype with upregulated levels of CD38 on CD8 and CD4 T cells is a well‐established predictive marker for disease progression.^74^ Additional activation markers upregulated during HIV infection include CD38/HLA‐DR coexpression on CD8 T cells and signs of ongoing replication as determined by Ki‐67+ T cells.^75^ T cells also exhibit impaired T‐cell maturation characterized by reduced expression of CD28 and high levels of CD27 costimulatory molecules, suggesting a decreased effector phenotype.^76^ Decreased levels of CD28 on HIV‐specific CD8 T cells are associated with shorter telomere lengths and reduced proliferation.^77^ HIV‐specific CD8 T cells with elevated levels of CD27 also have reduced Granzyme A and perforin cytotoxic activity compared to CD27 low effector T cells.^78^ Moreover, HIV‐specific memory CD8 T cells were also found to have a preterminally differentiated phenotype (CD45RA‐CCR7‐), when compared to CMV‐specific cells that instead expressed a terminally differentiated (CD45RA + CCR7‐) phenotype.^79^, ^80^

Cytokines including IL‐10 are also implicated in T‐cell exhaustion during HIV infection. IL‐10 production is part of the body's response to chronic inflammation established primarily through strong upregulation of the type I and II IFN‐related genes and pathways. Tregs accumulate at the sites of chronic HIV infection and play a direct role in the promotion of T‐cell exhaustion through production of IL‐10 that inhibits T‐cell proliferation.^81^ Aside from Tregs, multiple cell types contribute to IL‐10 production and patients with elevated plasma levels of IL‐10 correlate with rapid disease progression and impaired CD4 T‐cell help. In vitro blockade of IL‐10 increases proliferation of HIV‐specific CD4 and CD8 T cells and increases production of cytokines by CD4 T cells.^82^, ^83^

Overall, HIV‐specific T‐cell exhaustion established in the early stages of infection represents an almost insurmountable barrier to the immune mediate control of viral load and the elimination of HIV‐infected cells. Individuals who can spontaneously control HIV infection are rare, representing ≤1% of those infected.^84^ However, a recent longitudinal study provides evidence that T‐cell exhaustion plays an important role in the loss of viral control by these rare HIV controllers. These studies showed that just prior to increases in patient viremia, PD‐1 levels increased on HIV‐specific CD8 T cells and these cells exhibited reduced in vitro capacity to kill HIV‐infected cells.^85^ As such, a delicate balance may exist between T‐cell–mediated control of viral infection and the progressive development of T‐cell exhaustion.

## 7 T‐CELL EXHAUSTION IN OTHER VIRAL INFECTIONS

Pathogens have evolve through natural selection to evade immune‐mediated elimination by exploiting the IC pathways needed by the host to maintain peripheral tolerance and limits immunopathology under physiologic conditions. A key advantage in the discovering T‐cell exhaustion using the LCMV mouse infection model was the different viral laboratory strains and/or different viral inoculum that induce either an acute infection that could be resolved or a persistent chronic infection.^47^, ^48^, ^49^ Although CD8 T‐cell dysfunction was known to be an essential feature of exhaustion, the identification of PD‐1 as a key player during chronic infection was revealed much later through gene expression analysis. These studies showed that in the early stage of infection, PD‐1 was upregulated to similar levels in mice infected with either Armstrong (acute) or clone 13 (chronic) LCMV viruses. Clearance of Armstrong strain lead to a rapid downregulation of PD‐1 on virus‐specific CD8 T cells. However, in chronic clone 13 strain LCMV infection, virus‐specific CD8 T cells showed sustained increase PD‐1 levels that progressively lost their functionality. In this model, transient depletion of CD4 T cells has no effect on an acute viral strain that can be resolved within 2 weeks. However, in studies using a chronic LCMV variants that requires >3 months to be contained and cleared by the LCMV‐specific CD8 T‐cell response, even transient depletion of CD4 T cells at the time of infection resulted in a complete loss of CD8 T‐cell response to control the virus.^14^ These studies provide support for the concept that loss of CD4 T‐cell help can enhance conditions that lead to CD8 T‐cell exhaustion. An important concept validated in the LCMV model was that antibody‐mediated blockade of the PD‐1/PDL‐1 interaction reversed signature featured of T‐cell exhaustion that allowed for proliferation and increased functionality of LCMV‐specific T cells that lead to the killing of infected cells and decrease viral load.

Human cases of viral hepatitis have common features with LCMV infection where virus is either cleared by the immune system or leads to chronic infection in 10% and 70% of HBV‐ and HCV‐infected patients, respectively. The important role of virus‐specific CD8 T cells was demonstrated in non‐human primate models for HBV and HCV infection where depletion of CD8 cells lead to a prolonged viremia that only declined when CD8 T cells returned.^86^, ^87^ The two primary mechanism for chronic hepatitis infection in patients is viral mutation that leads to escape from antiviral CD8 T cells and through exhaustion where CD8 T cells lose their effector function. A strong and early CD4 T‐cell helper response in HCV infection is also associated with viral clearance in patients. In contrast, development of HCV‐specific CD4 T cells with limited proliferative potential at an early stage following infection resulted in the evolution of a chronic infection.^88^ Studies performed with blood mononuclear cells from chronically infected HBV patients showed that blockade of the PD‐1 pathway resulted in enhanced proliferation and functionality of HBV‐specific CD8+ T cells.^10^, ^89^ Furthermore, a HBV‐related chronic hepadnaviral infection model in woodchucks showed that PD‐1 blockade in combination with an antiviral agent and therapeutic DNA vaccination restored virus‐specific CD8+ T cells functionality and enhanced a continual immune‐mediated viral control.^90^ PD‐1 blockade alone had a moderate therapeutic result in patients with chronic HCV infection where 20% of those treated showed a significant drop in viral load.^91^ However, this result may be due to the nature of exhaustion associated with HCV since in vitro studies showed that hepatic PD‐1 + CTLA‐4 + virus‐specific CD8 + T cells where only functionally restored with a combination of CTLA‐4 and PD‐1 blockade and not with either therapy administered alone.^92^

Human T‐cell lymphotropic/leukemia virus type 1 (HTLV‐1) causes adult T‐cell leukemia/lymphoma and in both asymptomatic carriers and ATL patients there is an inverse correlation between HTLV proviral load and the functionality of CD8 T cells in response to viral antigens. Decreased functionality correlated directly with PD‐1 levels expressed on CD8 T cells and blockade through PDL‐1 significantly increased anti‐HTLV functionality. Of note, CD8 T‐cell functionality and levels of HTLV‐1 provirus did not correlate with expression levels of TIM3, LAG3, or CTLA‐4, indicating that different chronic viral infections or the extent of exhaustion may favor the upregulation of different ICI subsets.^93^, ^94^

## 8 MAJOR SIGNALING PATHWAYS INVOLVED IN T‐CELL EXHAUSTION

T‐cell activation takes place in a highly organized process where the antigenic peptide‐MHC complex on APCs binds to its cognate T‐cell TCR complex forming the core of the cell‐cell interaction. This primary contact sets into motion the reorganization of cell surface and cytosolic molecules leading to the formation of the immunological synapse. It is during this contact with the APC that ICIs exert their inhibitory effect to suppress signaling events needed for a fully activated T‐cell response.

Following immunological synapse formation, Lck phosphorylation of TCR‐associated CD3 ζ‐chains initiates the intracellular signaling cascades with ZAP‐70 propagating the TCR signal through phosphorylation of downstream intermediaries including PLC‐γ1.^95^, ^96^ T‐cell costimulation through CD28 is needed for robust, functional T‐cell response and acts through phosphatidylinositol 3‐kinase (PI3K) to activate the PI3K/AKT/mTOR signaling network. Both the TCR and CD28 costimulatory pathways converge to upregulate the activation of AP1, NFAT, and NFκB, which induce the transcription of T‐cell immune response genes such as IL‐2, IRF‐4, and pro‐survival factors.^97^ The strength, affinity, and duration of the initial TCR‐peptide‐MHC complex as well as the presence of costimulatory receptors dictate the level of response from the different signaling cascades and degree of their contributions on transcription factor activation.

ICIs exert their regulatory effect on T cells through a variety of mechanism. Several receptors including PD‐1 have intracellular domains containing immunotyrosine inhibitory motif (ITIM) and/or immunotyrosine switch motif (ITSM). These ITIM or ITSM motifs can recruit SHP tyrosine phosphatase proteins and other adapter into the vicinity of the TCR in order to disrupt positive signaling events. ICIs that suppress T‐cell activation through the ITIM/ITSM mechanism includes PD‐1, 2B4, LAIR‐1, and KLRG‐1.^98^ A second mechanism of T‐cell regulation through inhibitors receptors is by direct competition with costimulatory receptors. CTLA‐4 is the best studies example that can bind tightly to the CD80 and CD86 on APCs. In so doing, CTLA‐4 outcompetes CD28 for interaction with its natural ligands and increases the T‐cell activation threshold, especially for antigens that do not effectively induce T‐cell activation.^99^ ICIs including LAG3 and TIM3 have varied intracellular domains distinct from the classical ITIM/ITSM motifs and that recruit a different subset of molecules that are suppressive of T‐cell signaling. Finally, negative regulation can occur via receptors including TIGIT and BTLA that operate through a combination of different mechanism. TIGIT not only possess an intracellular ITIM motif on its cytoplasmic tail but also acts through a receptor competition mechanism in binding the CD155 and CD112 ligands on APCs that can activate T cells through interaction with CD226.^98^

PD‐1–mediated inhibition of T‐cell signaling is the best‐studied ICI pathway involved in T‐cell exhaustion. During T‐cell activation, the PD‐1 receptor migrates into T‐cell/APC contact region where it forms microclusters upon binding with PDL‐1. This recruitment brings the SHP2 and SHP1 phosphatases into membrane proximal region of the TCR and costimulatory receptors, inducing dephosphorylation of TCR‐linked signaling proteins CD3ζ, ZAP‐70, PLC‐γ1,^95^, ^96^ and the CD28 pathway involving PI3K and the associated downstream cascade through the AKT pathway. The PD‐1/SHP2 complex attenuates phosphorylation events associated with PKCθ, PI3K/AKT/mTOR, and Ras/MAPK/Erk signaling pathways needed for optimal T‐cell activation, proliferation, survival, and an altered cellular metabolism.^100^, ^101^ The dynamics of PD‐1 with CD28 and intracellular partners has been observed in fluorescence microscopy imaging studies where PD‐1 exists in microclusters on the cell surface and is recruited along with SHP2 phosphatase into the immunological synapse to suppress phosphorylation events during TCR activation.^3^, ^95^, ^96^, ^102^, ^103^, ^104^ One caveat with many of these mechanistic studies is that PD‐1 expression on T cells was achieved through prolonged in vitro T‐cell stimulation as opposed to exhausted T cells produced in vivo in response to chronic infection.

**Figure 4:**
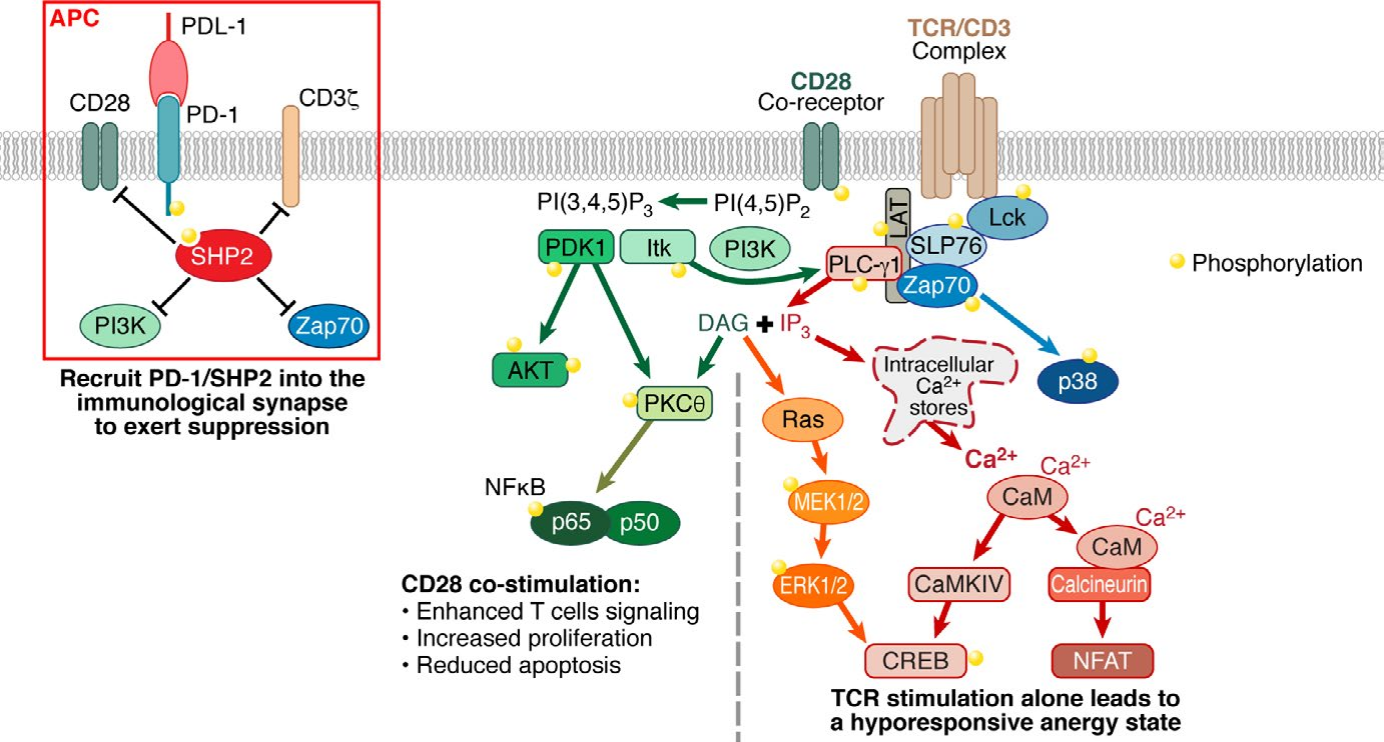
Signaling in T‐cell activation and PD‐1–mediated suppression. T‐cell activation through the TCR/CD3 complex leads to the phosphorylation of proximal signaling molecules that trigger downstream activation of NFAT, CREB, and p38 pathways. In the absence of a costimulatory signaling through CD28, TCR activation of T cells leads to a hyporesponsive, anergic state. Signaling through the CD28 costimulatory receptor enhances activation of the PI3K/AKT/NFκB pathways that support T‐cell proliferation and reduce apoptosis. Interaction of PD‐1 with PDL‐1 during T‐cell activation recruits the PD‐1/SHP2 tyrosine phosphatase complex into the vicinity of TCR/CD3 and the CD28 co‐receptor. This recruitment results in the dephosphorylation of membrane proximal PI3K, ZAP‐70 and the intracellular domains of CD28 and the CD3ζ chain, which suppresses T‐cell activation. In PD‐1 high exhausted T cells from chronically infected HIV donors, the ligation of PD‐1 with PDL‐1 results in reduced phosphorylation of TCR proximal Lck and Zap‐70 and downstream Erk1/2 in the Ras‐MEK1/2‐Erk1/2 pathway. PD‐1 suppression of T‐cell activation also results in reduced calcium mobilization that is upstream of CREB and NFAT transcription factors. In the CD28 costimulatory receptor pathway, ligation of PD‐1 with PDL‐1 significantly inhibits the PI3K/AKT/NFκB pathway with reduced phosphorylation of PDK1 and AKT

Our own laboratory recently reported a direct evaluation of signaling in truly exhausted T cells with elevated levels of PD‐1 from a chronically infected HIV donor.^105^ T‐cell activation in concert with PD‐1 ligation by PDL‐1 suppressed signaling event in both the TCR and CD28 pathways. TCR proximal phosphorylation of the Lck and ZAP‐70 was inhibited along with the downstream Ras‐MEK1/2 pathway monitored through Erk1/2 phosphorylation. Calcium flux experiments using exhausted T cells also showed a reduced degree of calcium mobilization in stimulations performed in the presence of PDL‐1, which leads to reduced activation of the NFAT pathway.^106^ The suppression of the CD28 signaling pathway resulted in significantly reduced phosphorylation of PDK1 and its direct target, AKT, phosphorylated at position T308. Formation of the full catalytically activation AKT, phosphorylated at S473 by mTORC2, was also inhibited in the exhausted T cells (Figure 4).

**Figure 5:**
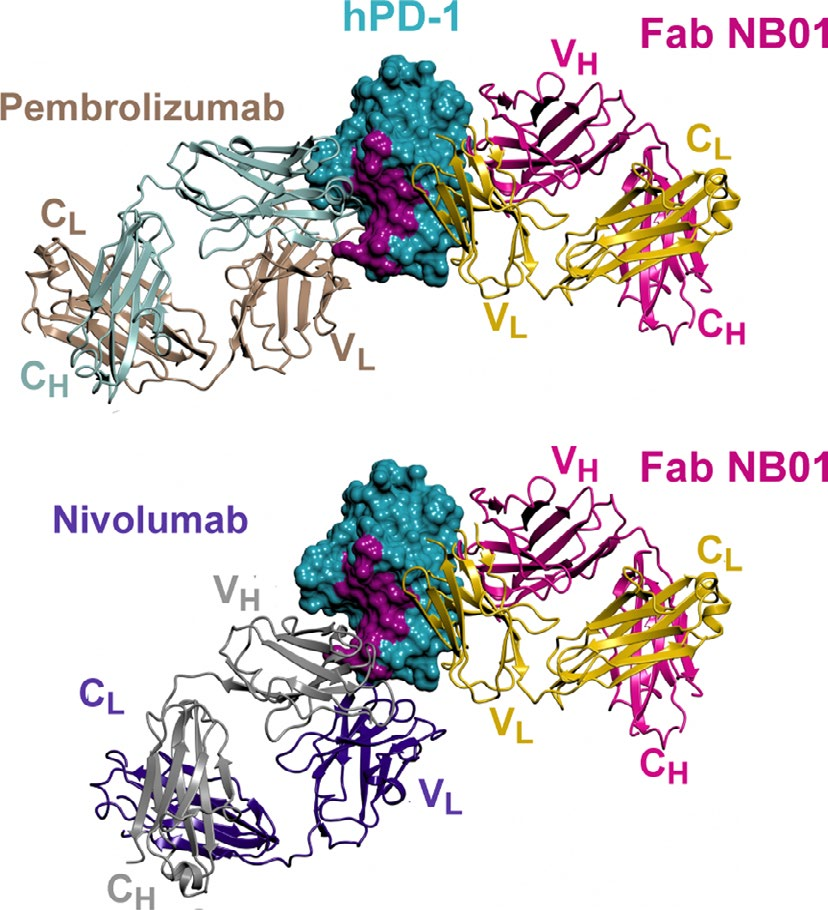
Antagonistic antibodies binding the PD‐1 receptor. The structure of human PD‐1 (hPD‐1) in complex with the anti‐PD‐1 NB01 Fab was solved by molecular replacement using crystals that diffracted to 2.2 Å resolution (6HIG). Molecular modeling by Cα superpositioning of hPD‐1 coordinates with the pembrolizumab (PDB 5GGS) and the nivolumab (PDB 5GGR) confirms that NB01 Fab binding to PD‐1 does not interfere with the binding of either pembrolizumab or nivolumab anti‐PD‐1 Abs. The hPDL‐1–binding surface (PDB 4ZQK) on hPD‐1 is colored in purple and is distinct from the binding epitope of the NB01 Fab that is non‐blocking of the PD‐1/PDL‐1 interaction

An important validation of the signaling pathways involved in PD‐1/PDL‐1–mediated T‐cell exhaustion is to monitor the restoration of specific phosphoprotein levels following anti‐PD‐1 therapy. In classical anti‐PD‐1 antibody therapy, relief of T‐cell exhaustion is achieved through PD‐1/PDL‐1 blockade. However, we recently reported the discovery and validation of a novel class of antagonistic anti‐PD‐1 antibody that are non‐blocking of the PD‐1/PDL‐1 interaction. Biochemical and structural studies demonstrated that these antibodies bound to the opposite face of the PD‐1 protein relative to the PD‐1/PDL‐1 interaction site. The region on PD‐1 targeted by these non‐blocking antibodies is highly conserved across six different species and may potentially represent a binding site for a yet to be identified alternate PD‐1 ligand or a region important for transmitting the negative regulatory effect of PD‐1. Structural modeling and competitive binding studies with cell surface PD‐1 also showed that the non‐blocking anti‐PD‐1 antibody NB01 could bind PD‐1 concomitantly with either pembrolizumab or nivolumab blocking anti‐PD‐1 antibodies (Figure 5). Consistent with blocking and non‐blocking antibodies both exerting distinct immune‐enhancing functional activity through PD‐1, treatment with combinations of the two antibody classes resulted in synergistic functional recovery of proliferation and IFN‐γ production from exhausted HIV‐specific CD8 T cells. In signaling studies performed in the presence of PD‐1/PDL‐1 suppression, both blocking and non‐blocking anti‐PD‐1 antibodies partially restored signaling phosphorylation of PDK1 and AKT at positions T308 and S473 in the CD28 pathway. These results are consistent with two recent studies showing that that anti‐PD‐1–mediated tumor suppressive activity is primarily dependent of the CD28 costimulatory receptor.^103^, ^104^ Both classes of anti‐PD‐1 antibodies also restored calcium mobilization that is downstream of the TCR activation pathway (Figure 4).

**Figure 6:**
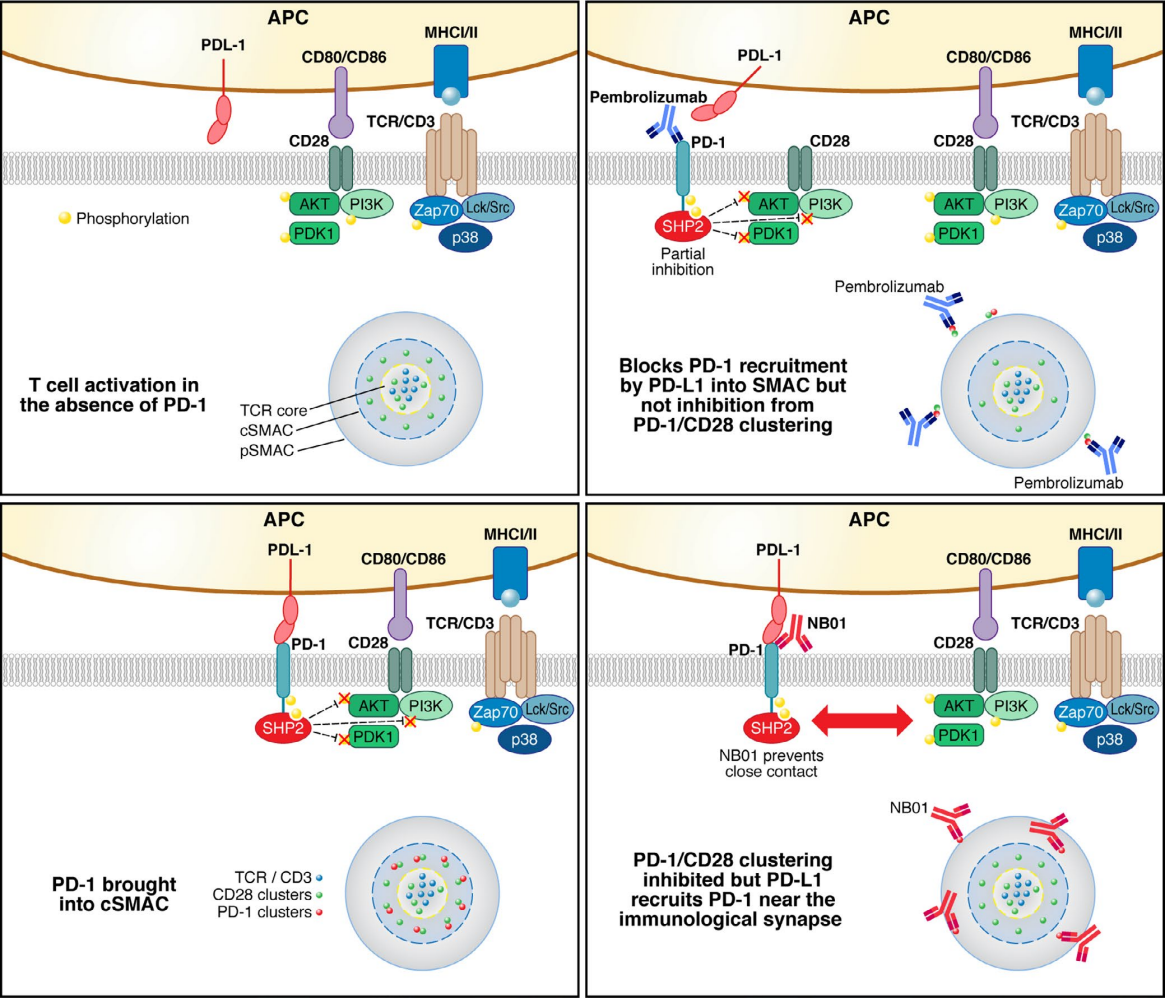
Anti‐PD‐1 antibody‐mediated restoration of exhausted T cells. (A) In the standard activation model of T cells expressing low levels of PD‐1, the immunological synapse forms with the TCR/CD3 complex at the core, surrounded by the CD28 costimulatory receptor within the cSMAC. This distribution forms a close complex of signaling molecules that enhances the T‐cell activation cascade. (B) In PD‐1 high T cells, PDL‐1 binding to PD‐1 recruits the PD‐1/SHP2 complex into the cSMAC that effectively suppresses signaling through the SHP2 phosphatase‐mediated dephosphorylation of TCR/CD3 and CD28 proximal signaling molecules. (C) Use of blocking anti‐PD‐1 antibodies such as pembrolizumab partially restores T‐cell signaling through limiting PD‐1/PDL‐1–mediated recruitment of the PD‐1/SHP2 complex into the cSMAC. The exclusion of SHP2 from the cSMAC reduces dephosphorylation of signaling molecules including ZAP‐70, Lck, PI3K, and AKT. However, our studies show that in activated T cells, pembrolizumab binding to PD‐1 pulls down a complex that includes SHP2, CD28, and PI3K following T‐cell activation. As such, PD‐1 may partially suppress T‐cell activation by recruiting CD28 away from the cSMAC and suppressing CD28 costimulation through the SHP2 phosphatase. (D) Our newly discovered anti‐PD‐1 antibody NB01 is non‐blocking of the PD‐1/PDL‐1 interaction and has equivalent antagonistic activity compared to pembrolizumab in restoring T‐cell signaling and antigen‐specific functional and proliferative activity to exhausted HIV‐specific T cells. Based on immunoprecipitation studies, our proposal is that non‐blocking anti‐PD‐1 antibodies act through inhibiting close contact of the PD‐1/SHP2 complex with CD28 and associated signaling molecules following T‐cell activation. As such, CD28 is free to migrate into the cSMAC and enhance T‐cell activation

Aside from the signaling relationship between PD‐1 and the CD28 pathway, immunoprecipitation studies performed with the pembrolizumab blocking anti‐PD‐1 antibody further demonstrated that in stimulated T cells, PD‐1 exists in a complex that includes CD28, SHP2, PI3K, and phosphorylated Lck Src protein. Importantly, our antagonistic non‐blocking anti‐PD‐1 antibody pulled down PD‐1 in complex with SHP2 and phosphorylated Lck Src, but showed significantly reduced interaction with CD28 and associated PI3K. This indicates that elevated levels of PD‐1 on a T cell may suppress T‐cell activation by two mechanism. One through PDL‐1–mediated recruitment of PD‐1 into the immunological synapse and other through a PD‐1 cis‐complex with the CD28 costimulatory receptor. The later colocalization would bring CD28‐associated intracellular kinases essential for T‐cell costimulation into contact with PD‐1–associated SHP2 phosphatase, resulting in a direct inhibition of the CD28 costimulatory signaling (Figure 6).

The coexpression of additional ICIs with PD‐1 in exhausted HIV‐specific T cells reflects an increased level of T‐cell dysfunction that is linked to the suppression of T‐cell signaling. These ICs include TIM3, TIGIT, and Lag3 that acting on distinct or overlapping points of the signaling cascade relative to PD‐1. TIM3 suppresses T‐cell activation through Lck, Fyn, and PI3K[107, 108 TIGIT recruits SHIP1 into the signaling complex which blocks further signal transduction to PI3K, MAPK pathways, and NFκB^109^ and LAG3 is structurally similar to the CD4 co‐receptor and acts through a poorly defined mechanism.^110^ With their combined inhibitory effect, it is not surprising that T cells with elevated levels of these ICIs are almost completely functionally defective and incapable of mounting an effective immune response.

## 9 RECOVERY OF T‐CELL FUNCTION: EFFECT OF ART

Upon initiating ART, the majority of patient have a dramatic reduction of viral load and HIV‐1 productively infected cells in both the periphery and lymph nodes.^111^ However, despite a spectacular efficacy in reducing morbidity and mortality associate with HIV infection, these drugs only inhibit viral replication and cannot cure those infected. ART maintains the level of plasma HIV‐1 RNA below the limit of detection for most treated patients. However, long‐lived latently infected memory CD4 T cells persist and the potential for residual virus replication prevent the eradication of infected cells.^112^ Even after decades of viral suppression, interruption of ART in patients invariably leads to a rapid viral rebound. In this regard, the continued immune dysfunction, while on ART is evident by the inability of the virus‐specific immune response to delay significantly the reemergence of the HIV virus. The major causes of this immune dysfunction in HIV are T‐cell exhaustion that prevents a functional control of the virus and necessitates life‐long ART in most infected patients to suppress viremia and prevent disease progression.

Patient under fully suppressive ART with undetectable HIV‐1 plasma viral loads gradual experience a downregulation of immune ICI expression on T cells, although frequencies remain higher compared to HIV‐uninfected individuals. A similar trend is observed CD38 + HLA‐DR + T cells, which represent an increased immune activation state.^113^, ^114^ The functionality of HIV‐specific T cells improves with ART treatment with both CD4 and CD8 T cells producing increased levels of IL‐2 following stimulation.^115^, ^116^ However, PD‐1, TIM3 and LAG3 expression on the T cells of ART‐treated patients was shown to be associated with the time to viral rebound in studies following standardized treatment interruptions.^117^ This demonstrates that the extent of T‐cell exhaustion contributes an underlying immune dysfunction that is unable to control even the low levels of virus produced from the latent HIV reservoir in the absence of ART. Our own unpublished results confirm a persistent functional exhaustion of HIV‐specific CD8 T cells in long‐term suppressive ART patients where PD‐1 blockade significantly restores IFN‐γ production by twofold. Similarly, combined blockade studies targeting TIGIT and PDL‐1 restored the proliferative capacity of virus‐specific CD8 T cells with enhanced proliferation compared to either single blockade.^58^

Early initiation of ART is clearly beneficial to patients on multiple levels. Early adoption of ART in the acute phase of viral infection correlates with a lower burden of latent HIV‐1 reservoir and reduced systemic inflammation.^118^, ^119^, ^120^, ^121^, ^122^ Since the development of T‐cell exhaustion is a progressive condition with hierarchical loss of functionality, early ART initiation represents the best opportunity to preserve a patient's HIV‐specific response before more pronounced and effectively irreversible T‐cell dysfunction is established.

## 10 INFLUENCE ON ICIS IN PERSISTENCE OF HIV

Multiple observational studies have demonstrated a clear association between expression of ICIs and the HIV reservoir. Amongst these, a pivotal study indicated that central memory CD4 T cells expressing PD‐1 were enriched in HIV‐infected cells, thus providing the first evidence for ICI expression on HIV‐infected cells.^123^ However, the first demonstration that PD‐1 expressing CD4 T cells might also be the source of replication competent virus was provided with the isolation and analysis of PD‐1^+^ and PD‐1^hi^/Tfh cells from subjects with non‐progressive infection and low viremia.^124^ Since then, many studies have shown a significant correlation between the frequency of PD‐1^+^ CD4 T cells with HIV persistence during ART in blood^125^, ^126^ and in tissues.^127^ However, the direct evidence of a clear relationship between HIV reservoir and PD‐1 expression came from isolated memory CD4 T cells from blood and lymph node of HIV‐infected aviremic ART‐treated individuals. These studies demonstrated that inducible replication competent HIV was found to be highly enriched in lymph node PD‐1^+^ CD4 T cells, containing the Tfh cell population.^128^ Recently, a further enrichment in HIV infection was shown in cells that express multiple ICIs simultaneously including PD‐1, TIGIT, and LAG3, suggesting that these inhibitory receptors not only suppress T‐cell activation but consequently suppress HIV transcription,^129^ and therefore favor HIV latency.^130^ These observations have prompted the investigation of whether ICI signaling through inhibition of T‐cell activation facilitate the establishment of latent HIV infection. Indeed, Evans *et al* recently demonstrated that PD‐1 blockade prior to in vitro HIV infection decreased the frequency of latently HIV‐infected cells in their in vitro model of HIV latency, highlighting the potential of ICIs blockade to disrupt latency.^130^ In addition, we recently observed that PD‐1/PDL‐1 interactions strongly inhibited TCR‐mediated reactivation of HIV transcription and viral production from lymph nodes memory CD4 T cells. Furthermore, PD‐1 blockade with anti‐PD‐1 monoclonal antibody treatment reactivated HIV replication from primary latently infected cells in vitro.^131^ These illuminating results revealing the association between HIV persistence and ICIs expression are now being further explored in in vivo studies in individuals with HIV and cancer. Several case report studies tested the potential benefit of using ICI blockers, that is, anti‐PD‐1 or anti‐CTLA‐4 monoclonal antibodies to (a) potentially reverse HIV latency in CD4 T cells, thereby allowing the expression of HIV proteins on the cell surface and to (b) reinvigorate HIV‐specific CD8 T cells from their exhausted state to potentiate the elimination of reactivated HIV‐infected cells. While several reports highlighted a potential reactivation of HIV reservoir markers,^132^, ^133^, ^134^ only one study reported a subsequent decrease in HIV reservoir size.^132^ Taken together, these revelations highlighted the enrichment of HIV replication competent virus within ICIs expressing CD4 T cells. Further investigation is needed to determine if targeting these T cells and relieving exhaustion could break latency and eliminate the HIV reservoir.

## 11 EXPLOITING PD‐1 TARGETING TO PURGE THE HIV RESERVOIR

Immunotherapy through PD‐1 blockade represents a major breakthrough that has provided a significant clinical benefit to patients for the treatment of different cancers.^135^, ^136^, ^137^ In vitro studies using the cells of HIV‐infected patients have established a clear proof of principle benefit in using anti‐PD‐1 or PDL‐1 antibodies to relieve exhaustion and enhance HIV‐antigen‐specific functionality and proliferation. Our own in vitro studies show that the combination of classical blocking anti‐PD‐1 antibodies with novel antagonistic anti‐PD‐1 antibodies that are non‐blocking of the PD‐1/PDL‐1 interaction synergize to relieve functional exhaustion of HIV‐specific CD8 T cells and represent an exciting option for HIV immunotherapy.^105^ In vivo PD‐1 blockade studies with SIV‐infected macaques demonstrated a rapid expansion and functional quality of virus‐specific CD8 T cells in both the blood and gut tissue. PD‐1 blockade reduction of plasma viral load and impressively prolonged the survival of SIV‐infected macaques.^138^ Anti‐PD‐1 therapy combined with ART vs ART alone in SIV‐infected monkeys also had a more rapid suppression of viral loads and delayed rebound after a standardized treatment interruption.^139^ Despite the success of these studies and others at boosting the immune‐mediated antiviral activity, SIV‐infected monkeys were not able to maintain immunological control of the SIV virus. As such, relieving T‐cell–mediated exhaustion through anti‐PD‐1 blockade is unlikely to be successful as a monotherapy. Although results are preliminary for several clinical studies employing PD‐1 blockade, the patients tested thus far have only shown a modest response at best.^132^, ^133^, ^134^ This indicates that immunotherapy targeting several ICIs in combination with other strategies to reactivate the virus from latently infected cells may be needed to purge the HIV reservoir.

**Figure 7:**
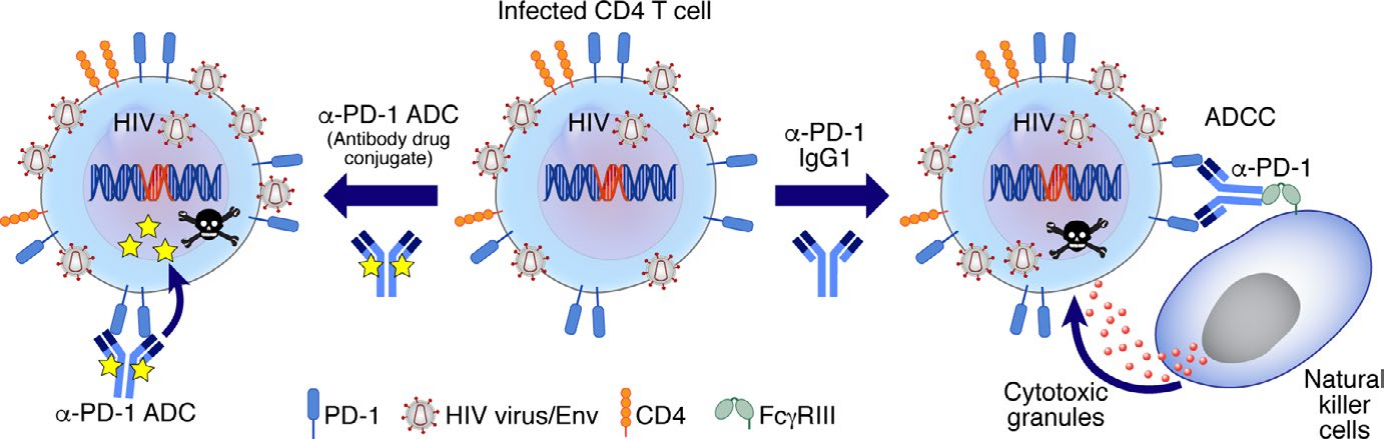
Targeted killing of PD‐1–positive HIV‐infected cells. PD‐1 expressing CD4 T cells represent the primary compartment of HIV‐infected cells that produce replication competent virus. Targeted killing of these PD‐1 positive cells represents a novel therapeutic strategy that can deplete the HIV reservoir of infected cells. Antibody drug conjugates (ADC) use a toxin‐conjugated antibody that binds to the cell surface PD‐1 receptor. Internalization of PD‐1 results in ADC degradation within the lysosome, resulting in toxin release that specifically kills the PD‐1 expressing cell. An alternate strategy for the targeted killing of HIV infected PD‐1 positive cells is through antibody‐dependent cellular cytotoxicity (ADCC) with an IgG1 anti‐PD‐1 antibody. The Fc portion of the antibody binds to the FcγRIII receptors expressed on effector cells including natural killer (NK) cells. NK cells release cytotoxic granules that kill the PD‐1–positive HIV‐infected CD4 T cell

The HIV virus has developed a considerable stealth in evading detection from a patient's immunological response. Antibody‐mediated immunotherapy targeting ICIs can address T‐cell functional exhaustion. However, a limitation is the lack of access of HIV‐specific cytotoxic CD8 T cells to privileged anatomic compartments including lymphoid organs where persistent viremia and/or residual virus replication may occur in memory CD4 T cells.^140^, ^141^ Approaches for the targeted killing of infected cells would provide an orthogonal method of eliminating the highly heterogeneous latent population of infected cells. Passive immunization using broadly neutralizing antibodies (bNabs) against the HIV‐1 Envelope protein may contribute to the killing of infected cells through antibody‐mediated effector function. However, a recent clinical study was unable to show a benefit in reducing HIV‐1 persistence in ART suppressed patients with a combined bNab therapy.^142^ A strategy currently under evaluation by our group exploits the fact that PD‐1 + CD4 T cells from blood and lymph nodes represent the major cell reservoir for replication competent and infectious HIV in chronic and in long‐term antiretroviral‐treated subjects. An anti‐PD‐1 antibody drug conjugate (ADC) was developed with a PNU toxin and in vitro studies show specific induction of apoptosis and cell death in PD‐1 positive cells. Anti‐PD‐1 ADC treatment of CD4 T cells from chronically infected HIV‐1 donors significantly reduced viral production relative to a control anti‐PD‐1 antibody. This therapy was also effective in aviremic ART donors, purging the majority of cells from that were capable of producing infectious virus (Figure 7).^131^ Although these data are very encouraging, a primary consideration with all of therapeutic approaches to eliminate the HIV reservoir will be safety. Considerable strides have led to the development of highly potent ART that effectively suppressed viral loads in most patients for decades with limited adverse events and liabilities. As such, the bar will be set high to demonstrate a clear medical need for the use of curative strategies that present any dangers to the long‐term health of patients.

## CONFLICT OF INTEREST

All authors declare no conflict of interest with this review.
